# Time-trend analysis of tuberculosis diagnosis in Shenzhen, China between 2011 and 2020

**DOI:** 10.3389/fpubh.2023.1059433

**Published:** 2023-02-20

**Authors:** Chuang-Yue Hong, Fu-Lin Wang, You-Tong Zhang, Feng-Xi Tao, Le-Cai Ji, Pei-Xuan Lai, Ming-Zhen Li, Chong-Guang Yang, Wei-Guo Tan, Qi Jiang

**Affiliations:** ^1^Department of Tuberculosis Prevention and Control, Shenzhen Center for Chronic Disease Control, Shenzhen, Guangdong, China; ^2^Department of Epidemiology and Biostatistics, School of Public Health, Wuhan University, Wuhan, Hubei, China; ^3^School of Public Health (Shenzhen), Sun Yat-sen University, Shenzhen, Guangdong, China

**Keywords:** tuberculosis, diagnosis delay, time trend, molecular diagnosis, risk factor

## Abstract

**Objective:**

To describe the trend of tuberculosis (TB) diagnosis in the migrant city Shenzhen, China, and analyze the risk factors of diagnosis delays.

**Methods:**

Demographic and clinical information of TB patients from 2011 to 2020 in Shenzhen were extracted. A bundle of measures to enhance TB diagnosis had been implemented since late 2017. We calculated the proportions of patients who underwent a patient delay (>30 days from syndrome onset to first care-seeking) or a hospital delay (>4 days from first care-seeking to TB diagnosis). Multivariable logistic regression was used to analyze the risk factors of diagnosis delays.

**Results:**

During the study period, 43,846 patients with active pulmonary TB were diagnosed and registered in Shenzhen. On average, the bacteriological positivity rate of the patients was 54.9%, and this increased from 38.6% in 2017 to 74.2% in 2020. Overall, 30.3 and 31.1% of patients had a patient delay or a hospital delay, respectively. Molecular testing significantly increased bacteriological positivity and decreased the risk of hospital delay. People >35 years old, the unemployed, and residents had a higher risk of delays in both patient care-seeking and hospital diagnosis than younger people, workers, or migrants. Compared with passive case-finding, active case-finding significantly decreased the risk of patient delay by 5.47 (4.85–6.19) times.

**Conclusion:**

The bacteriological positivity rate of TB patients in Shenzhen increased significantly but the diagnosis delays were still serious, which may need more attention when active case-finding in risk populations and optimization of molecular testing.

## 1. Introduction

China has the second highest tuberculosis (TB) burden in the world. In 2020, it is estimated that the number of new TB cases in China was 842,000, with an incidence rate of 47.8/100,000 (1). Delays in the diagnosis of TB often lead to exacerbations, increased mortality, and further community transmission (2). The lack of bacteriological tests and drug-resistance screening, which is the gold standard for the diagnosis of pulmonary TB, is an important cause of the diagnosis delay (3). The commonly used sputum smear method has low sensitivity (40–0%) and the period for sputum culture is long (4–8 weeks), while molecular diagnostic technologies, such as GeneXpert, not only improve the detection sensitivity (>90%) but also shorten the detection time (0–1 days) (4). With the improvement of diagnostic technology, the time of diagnosis and risk of delay have both decreased (5), but the limited use in primary care makes effective TB diagnosis challenging.

Shenzhen is one of the most economically developed cities in China, with the majority of the inhabitants being internal migrants from other areas of China. Measures to enhance TB diagnosis, primarily the application of molecular testing, have been promoted in primary care in Shenzhen since late 2017 (6). Since then, the diagnosis of TB patients has improved markedly; for example, the sputum culture rate of pulmonary TB patients in the Futian District increased from 53.9% before 2017 to 94.5% in 2018 (7). However, the changes in the diagnosis delay of TB before and after the application of molecular testing are still unclear. In this study, we describe the changes in TB diagnosis and analyze the risk factors of diagnosis delays among both migrants and residents in Shenzhen from 2011 to 2020 to provide an updated description of TB diagnosis and optimized recommendations for TB control strategies.

## 2. Methods

### 2.1. Study setting and data collection

Shenzhen is one of the most economically developed cities in China with a per capita GDP of $27,000 in 2021 (8). It has 10 administrative districts under its jurisdiction, with a permanent population of nearly 20 million, of which more than two-thirds are internal migrants (8). Since the fourth quarter of 2017, a bundle of measures to enhance TB diagnosis were promoted in Shenzhen, particularly the use of the molecular testing methods GeneXpert (Cepheid, CA, USA) or EasyNAT (Ustar, Hangzhou, China) in primary care (6). To increase the culture-based testing of drug susceptibility, the proportion of sputum culture testing among TB patients was requested to be increased to more than 95% (9). In addition, patients with negative bacteriological results were diagnosed in strict accordance with the clinical diagnosis guidelines and with the consultation and discussion of at least three chief physicians.

Data of TB patients from 2011 to 2020 in Shenzhen were extracted from the National Tuberculosis Registration System. We collected the demographic and clinical characteristics of the patients, including gender, age, ethnicity, occupation, TB history, way of case-finding, bacteriological testing results, drug resistance, and treatment outcome. Patients with simple TB pleurisy or extrapulmonary TB and patients with missing key information were excluded from the analysis.

### 2.2. Critical definitions

Active pulmonary TB patients were diagnosed based on the National Guidelines for TB Diagnosis (6) in both inpatients and outpatients. Suspected patients provided three sputum specimens for bacteriological confirmation, and bacteria-positive patients were confirmed if at least one of either sputum smear, culture, or molecular testing was positive. Drug-resistant TB occurred when a patient infected with *Mycobacterium tuberculosis* developed resistance to one or more anti-TB drugs. The diagnosis delay was the time interval between the onset of TB symptoms and diagnoses. Considering the different factors affecting the patient and hospital, the interval of the diagnosis delay was divided into two parts—the delay in the care-seeking of the patient (patient delay) and the diagnosis in the hospital (hospital delay). A patient delay occurred if the interval between the onset of symptoms and the first care-seeking was more than 30 days, and a hospital delay occurred if it took more than 14 days from the patient's first care-seeking to diagnosis (3, 10). In terms of case-finding, ‘active case-finding' included X-ray screening of TB close contacts and physical examinations of employees or elderly people, whereas ‘passive case-finding' included care-seeking of symptomatic patients and referrals from primary care.

### 2.3. Statistical analysis

The data were collected and cleaned in Excel. The incidence was calculated by dividing the reported cases per year by the average population during the study period. The proportions of patients with a patient delay and hospital delay were calculated and visualized as time-trend curves. The trends were tested using a chi-square trend test. Differences in category variables between groups were compared to screen the related factors of diagnosis delay using a chi-square test. Variables with a *p*-value < 0.2 were included in the multivariable logistic regression model to estimate the adjusted odds ratios (aOR) with the 95% confidence intervals (CI) of the independent risk factors. The difference was considered to be statistically significant when *p*<0.05. All the statistical analysis was performed in R (v4.1.3).

## 3. Results

### 3.1. Basic information about the patients

**Figure 1:**
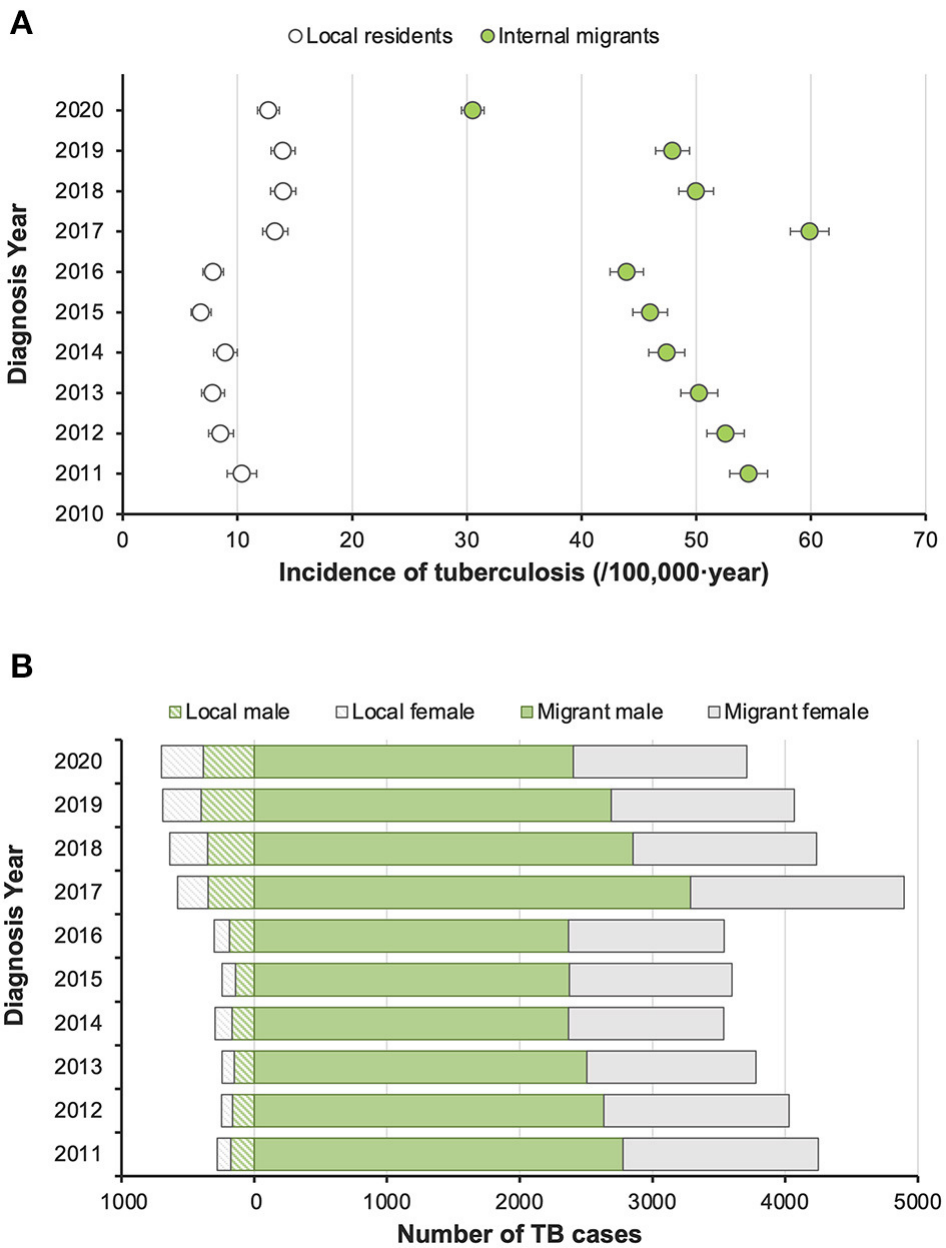
Trends of reported incidence of pulmonary tuberculosis patients in Shenzhen and their distribution by gender and population category from 2011 to 2020. **(A)** Reported incidence of active pulmonary tuberculosis by year. **(B)** Gender composition of diagnosed TB patients in local and migrant populations by year.

From 2011 to 2020, 43,846 active pulmonary TB patients were registered in Shenzhen, and the number of registered patients was 3,800–5,500 per year. Overall, the reported incidence was 30.2 (95% CI 29.9–30.5) per 100,000 population per year. Internal migrants accounted for 90.4% (39,641/43,846) of the patients and had a significantly higher reported incidence than the local residents, with an incidence of 37.1 (36.7–37.4) and 11.0 (10.6–11.3) per 100,000 population per year, respectively (Figure 1). Before 2017, the overall reported incidence in both migrants and residents showed a slow downward trend, but since 2017, improvements in TB diagnostic techniques and strategies led to a marked increase in reported incidence. Among all the patients, males accounted for 65.5% (28,730/43,846), and most patients (66.3%, 29,079/43,846) were under the age of 35. Migrant patients and local residents in Shenzhen were quite different in terms of demographic characteristics but they showed similar clinical features of TB, including bacteriological confirmation and disease severity (Supplementary Table 1).

### 3.2. Bacteriological confirmation

**Figure 2:**
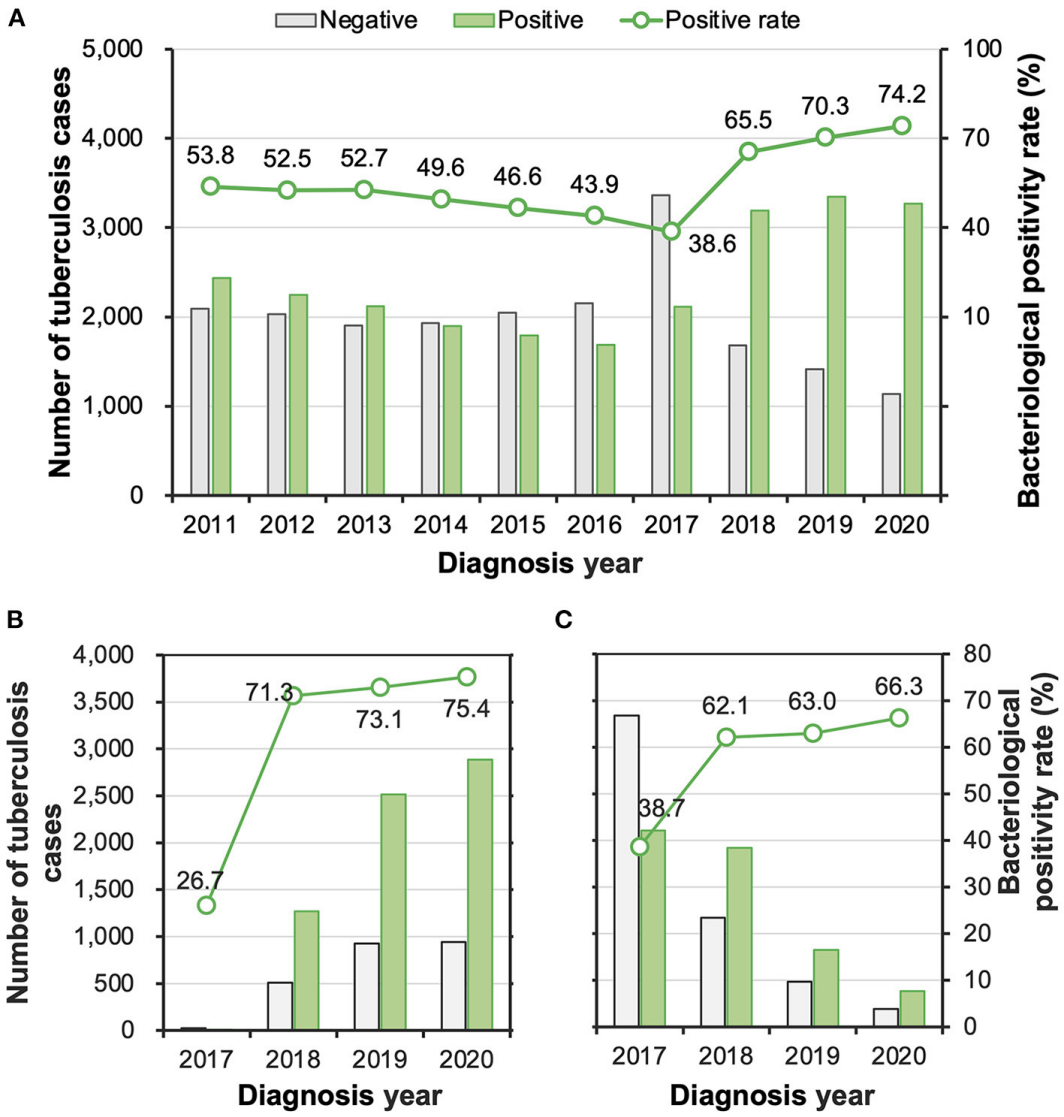
**(A–C)** The numbers and proportions of tuberculosis patients with and without bacteriological confirmation overall **(A)** among patients who took molecular tests **(B)** and among patients who did not take a molecular test **(C)**.

During the study period, 54.9% (24,094/43,846) of the patients were diagnosed with pulmonary TB with bacteriological confirmation. The rate decreased from 53.8% (2,434/4,526) in 2011 to 38.6% (2,112/5,473) in 2017 but increased sharply to 65.5% (3,190/4,871) in 2018, and then continued to rise to 74.2% (3,270/4,409) in 2020 (Figure 2). The change in the diagnosis of bacteriologically confirmed TB might have partially resulted from the promotion of molecular testing, which was performed for 36.5% (1,778/4,871) of the patients in 2018 with a remarkable increase to 86.9% (3,831/4,409) of the patients in 2020. Among the patients who receive a molecular test, 73.5% (6,680/9,083) were diagnosed with bacteria-positive TB, while the rate was significantly lower among patients who did not receive a molecular test (Figure 2).

### 3.3. The trend of diagnosis delays

**Figure 3:**
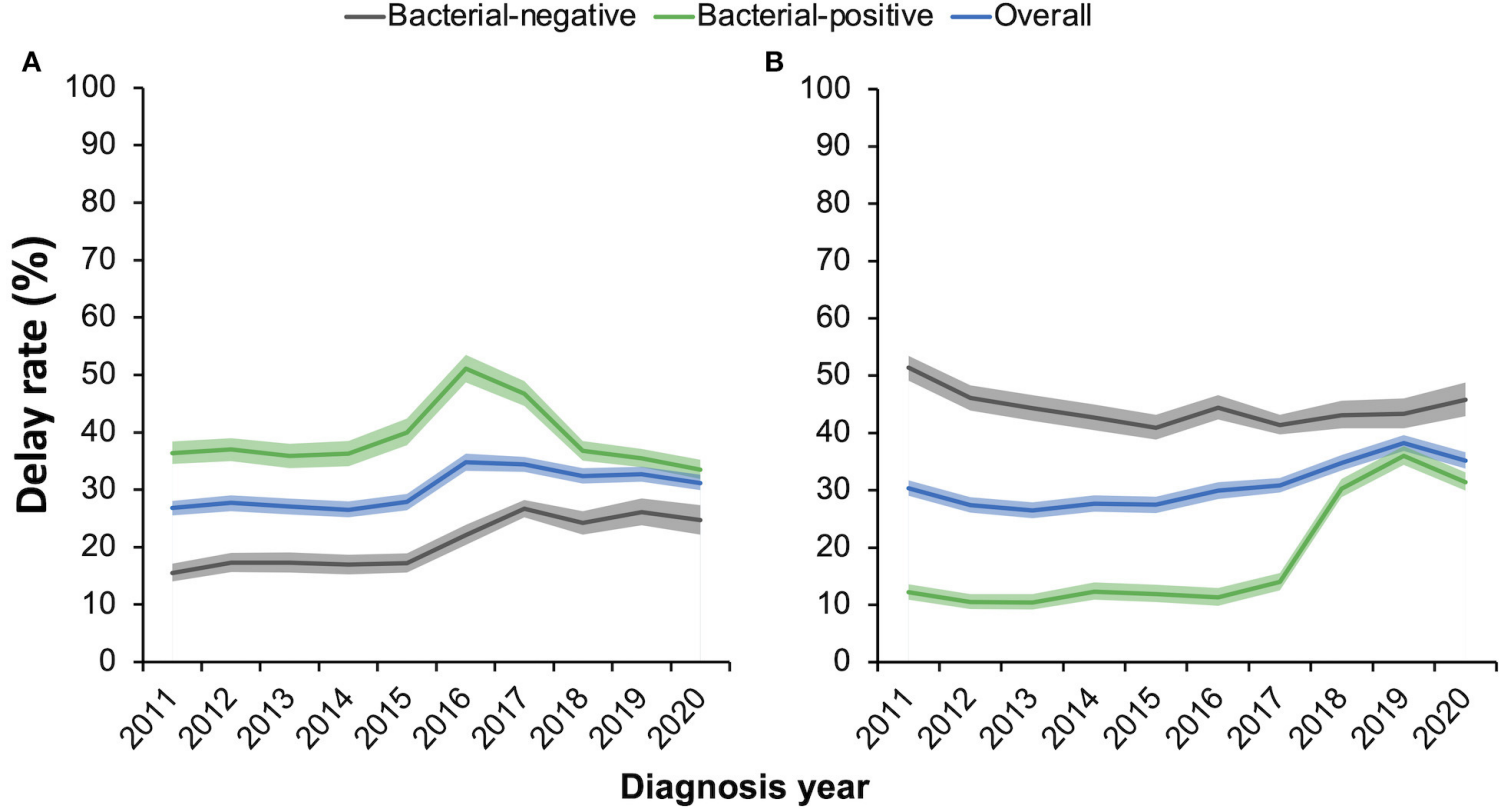
**(A, B)** Proportions of pulmonary tuberculosis patients experiencing a patient care-seeking delay of >30 days **(A)** or a hospital-diagnosis delay of >14 days **(B)**. Shaded areas show the 95% confidential intervals of the estimates in each year.

To clarify whether the diagnostic-enhancing measures reduced the diagnosis delay of TB patients, we estimated by year the proportions of patients who underwent a delay of >30 days in patient care-seeking or a delay of >14 days in hospital diagnosis (Figure 3). Overall, 30.3% (13,305/43,846) and 31.1% (13,628/43,846) of the patients had a patient delay and/or a hospital delay, respectively. Of these, 6.2% (2,721/43,846) had delays in both patient care-seeking and hospital diagnosis. Generally, more bacteria-positive patients had delays in care-seeking than bacteria-negative patients, while they were less likely to experience a hospital delay. The patient delay rate of bacteria-positive patients appeared at a steady level of 37% before the year 2014, slightly increased between 2014 and 2016, and then continued to decrease after 2017. Nevertheless, they had a relatively low level of hospital delay, which slightly fluctuated around 12% before 2017 and rapidly rose to 30% between 2018 and 2020. This rise in the hospital delay rate might have resulted from the lengthy duration of culture testing (2–4 weeks) for patients who waited for the last bacteriological testing after negative results from sputum smear or molecular testing. Comparably, hospital delay remained at a high level for bacteria-negative patients, ranging from 40 to 50% during the study period.

### 3.4. Risk factors of diagnosis delays

Given that the reasons for the patient delay and hospital delay might be different, we compared the demographic and clinical characteristics of patients with and without these two kinds of delays. In terms of patient delay, significant differences were observed in the distribution of characteristics between delayed and non-delayed patients, including gender, age, occupation, way of case-finding, chest radiographic cavity, and drug resistance type (Supplementary Table 2). In the multivariable logistic regression model, independent risk factors for patient delay included females (aOR 1.31, 95% CI 1.25–1.37), age >35 years (aOR 1.35, 95% CI 1.29–1.41), unemployment (aOR 1.18, 95% CI 1.14–1.24), local residents (aOR 1.12, 95% CI 1.04–1.20), and passive case-finding (aOR 5.47, 95% CI 4.85–6.19) (Table 1). Active case-finding could effectively decrease patient delay; however, it only detected 9.9% (4,338/43,846) of all the patients, including 2.2% of delayed patients and 13.2% of non-delayed patients (Supplementary Table 2). Retreated patients were less likely to have delays in patient care-seeking, while delayed patients were prone to being bacteria-positive, having a chest radiographic cavity, or being drug-resistant.

**Table 1:** Risk factors for patient delay and hospital delay identified by multivariable logistic regression.

	**Patient delay**		**Hospital delay**	
	**aOR (95% CI)**	***P-*value**	**aOR (95% CI)**	***P-*value**
Female	1.31 (1.25–1.37)	< 0.0001	1.00 (0.95–1.04)	0.9186
Age >35 years	1.35 (1.29–1.41)	< 0.0001	1.03 (0.98–1.08)	0.1887
Unemployed	1.18 (1.14–1.24)	< 0.0001	0.89 (0.85–0.93)	< 0.0001
Local resident	1.12 (1.04–1.20)	0.003	1.17 (1.09–1.25)	< 0.0001
Retreated patient	0.72 (0.65–0.78)	< 0.0001	1.19 (1.07–1.32)	0.0009
Passive case-finding	5.47 (4.85–6.19)	< 0.0001	0.24 (0.23–0.26)	< 0.0001
Bacteria positivity	2.03 (1.94–2.13)	< 0.0001	0.33 (0.31–0.34)	< 0.0001
Radiographic cavity	1.34 (1.27–1.40)	< 0.0001	0.65 (0.61–0.69)	< 0.0001
Drug resistance	1.12 (1.01–1.23)	0.024	0.88 (0.78–0.99)	0.0354
Intervention period (2018–2020)	0.90 (0.86–0.94)	< 0.0001	2.01 (1.92–2.11)	< 0.0001

aOR, adjusted Odds Ratio; CI, Confidential Interval.

Similarly, local residents (aOR 1.17, 95% CI 1.09–1.25) also had a higher risk of a delay in hospital diagnosis than migrants (Table 1; Supplementary Table 3). Unlike patient delay, retreated patients were more likely to have a hospital diagnosis delay, while passive case-finding patients had a lower risk than active case-finding patients. Patients with bacteria positivity, a chest radiographic cavity, or drug resistance appeared to be diagnosed more easily in the hospital and presented a decreased risk.

During the intervention period, the risk of patient delay was reduced (aOR 0.90 [95% CI 0.86–0.94]), whereas the risk of hospital diagnosis delay increased by 2.01 times (95% CI 1.92–2.11). Among patients during the intervention time, the use of molecular testing significantly decreased the risk of hospital delay by 45% (Supplementary Table 4). Risk factors between local residents and internal migrants were generally similar, except that more migrants delayed in care-seeking were unemployed or drug-resistant than residents (Supplementary Table 5). Active case-finding showed more efficiency among residents than among migrants, with a decreased risk of patient delay of 8.03 (5.53–12.14) and 5.23 (4.61–5.96), respectively.

## 4. Discussion

Based on the systematic registration data of TB patients in Shenzhen from 2011 to 2020, we described the trend and change in TB diagnosis and analyzed the risk factors for delays in patient care-seeking and hospital diagnosis. The reported incidence in both internal migrants and local residents generally showed a gradual decrease, except for a small jump in 2017 when measures to enhance TB diagnosis were promoted in Shenzhen. The measures significantly increased the bacteriological positivity rate of TB patients, which increased from 38.6% in 2017 to 74.2% in 2020. A total of 30.3 and 31.1% of the patients underwent a delay of >30 days in patient care-seeking and a delay of >14 days for hospital diagnosis, respectively. The proportions of patients with care-seeking delay dropped back to the initial level after an increase between 2014 and 2016, while hospital delay significantly increased in bacteria-positive patients after 2017. Local residents had a higher risk of delays in both patient care-seeking and hospital diagnosis than migrants, and active case-finding reduced TB diagnosis delays in both populations.

During the study period, the average bacteriological positivity rate of patients with active pulmonary TB was 54.9%. The rate increased sharply from 38.6% in 2017 to 65.5% in 2018 and then continued to rise to 74.2% in 2020. The significant change was thought to be closely related to the change in TB diagnosis strategies in Shenzhen. According to the national TB diagnosis guidelines (6), a bundle of measures to enhance TB diagnosis was implemented in Shenzhen in late 2017. First, indicators of bacteriological confirmation were requested, such as >95% of the patients being tested by sputum culture and >50% of patients being bacteriologically confirmed. The recent bacteriological positivity rate of TB patients in Shenzhen was much higher than the requested level and the average level in China (30–40%), and very close to the levels in developed European countries (1).

Besides, TB control programs increased the economic support of molecular testing technologies, which were quickly implemented in the primary care of TB diagnosis in Shenzhen. The proportion of patients taking a molecular test increased from 36.5% in 2018 to 86.9% in 2020. Although not all patients were tested by molecular methods, those that were had a higher rate of bacteriological positivity, and the annual rate of bacteria-positive TB was obviously increased during the intervention period. Studies have shown that the application of GeneXpert can accurately screen patients in high-burden settings and significantly shorten the detection time of both bacteria-positive and drug-resistant tuberculosis (11, 12). However, the implementation of the technology in primary care was limited due to the high cost, especially in rural areas (13). The local product EasyNat has a lower cost and does not require any equipment but cannot be used to test for drug resistance (14). Combinations of molecular testing of drug resistance, such as the melting curve method or whole-genome sequencing, could complement the testing pipeline of TB diagnosis (15). To conclude, the implementation of multiple TB diagnostic technologies still needs more investment and improved strategies to meet the national requirements in various regions of China.

Using an absolute threshold to define the delays in patient care-seeking and hospital diagnosis, we estimated that approximately one-third of the TB patients experienced delays in patient care-seeking or hospital diagnosis. The thresholds were close to the median durations of patient delay (28 days [95% CI 20–30]) and hospital delay (14 days [95% CI 2–28]) in a meta-analysis (16). Some studies in China defined the patient delay of over 2 weeks from the syndrome onset to the first care-seeking and estimated 46.4 and 59.0% of the TB patients had delays in Beijing (17) and Hubei (18), respectively. The levels were slightly lower than that of Shenzhen-−59.0% under the 2-week threshold. However, the rate of hospital diagnosis delays in Shenzhen was much lower than the national average level of 57.3%, as reported in a retrospective cohort study of multiple sites in China (3). Patient delay decreased but hospital delay increased during the intervention period of TB diagnosis enhancement (2018–2020). The former effect might have resulted from the increased reputation of the medical institutions and propaganda about TB diagnosis and treatment. The latter could be influenced by the longer hospital delays in bacteria-positive patients, as shown in Figure 3.

Patients with care-seeking delays appeared more likely to be bacteria-positive, have cavities in chest radiographs, and be drug-resistant. The delays in care-seeking might increase the bacterial load of the patients and cause more severe disease (10). Patients with more severe diseases had obvious symptoms or typical signs on the chest radiograph and were more likely to test bacteria-positive, and thus, they were less likely to experience a hospital delay. As shown in Figure 3, bacteria-negative patients had a relatively high level of hospital delay of 40–50%, whereas only ~10% of the bacteria-positive patients had a hospital delay before 2017. The rate of hospital delay among bacteria-positive patients increased to ~30% between 2018 and 2020, which was thought to result from the delayed diagnosis of patients who had only tested positive with cultures. Patients with negative molecular testing results or sputum smear would wait for the results of culture testing and then consult the physician, which took a long time but may have been more accurate. The study in Ethiopia also showed that smear-negative patients had longer health system delays (19). Although patients who had only tested positive with cultures had longer hospital delays, the diagnosis-enhancing measures, which mainly included the application of molecular testing, significantly increased the proportion of bacteria-positive TB patients and resulted in more accurate TB diagnoses.

Passive case-finding patients had more than five times the risk of delayed in care-seeking than active case-finding patients. A study across multiple sites in China showed the pathway of TB diagnosis, which always involved multiple medical providers during the transfer of patients (3). Similar to most areas in China, active case-finding only detected a relatively limited number of TB patients. Active case detection through varied strategies has been shown to significantly improve case-finding and reduce diagnosis delays (20, 21). For example, community-based active case-finding by screening TB in high-burden groups, such as the elderly or people with diabetes, could have a long-term health impact (22, 23). Besides, a study in Mumbai suggested that immediate TB testing by fully qualified providers at the patient's first visit could reduce the average diagnosis delay from 23.39 days to 11.16 days (24).

In our dataset, it was also found that females, patients >35 years old, and the unemployed had a higher risk of care-seeking delay than males, younger patients, and workers. The results were similar to a meta-analysis involving 19 low- and lower-middle-income countries, which found a 1.48 times (95% CI 1.09–1.98) higher risk of diagnosis delays in females than in males and a 2.70 times (95% CI 1.27–5.83) higher risk in patients >55 years old than in younger people (16, 25). This tendency might be a result of lower awareness and willingness to seek medical care in risk populations. Additionally, elderly patients were more likely to experience delayed hospital diagnosis (20). This result suggested that TB prevention and control should pay more attention to these risk populations, especially females and elderly people. Another study (26) in Tanzania found that gender and age were not risk factors for diagnosis delays, probably because the diagnosis delay was calculated by summing up the delays from both the patient and hospital. In Ethiopia, new cases had nearly three times the risk of delayed care-seeking than retreated patients, which might be due to the limited amount of information about TB before diagnosis and treatment (27). Surprisingly, migrants had a lower risk of patient delay than local residents in our dataset. Migrant patients were thought to have more barriers to medical care, such as the inconvenience of medical insurance reimbursement and lack of time or social support (28). They were shown to have a longer delay in both patient care-seeking and hospital diagnosis in an eastern county of China (29). Active case-finding was less efficient among migrants than among residents, which also reminded us that the healthcare quality of migrants should be improved through multidimensional social support (30).

This study had several limitations. First, the diagnosis delays were analyzed as binomial variables instead of continuous data, which might affect the accuracy of the results. Second, as TB infection does not always have typical symptoms, the self-reported date of the symptom onset could be subjective and might have reporting bias in the patient delay. Third, the logistic regression models suggested risk factors for delayed TB diagnosis but were limited in explaining its causality, and more investigation is needed.

In conclusion, after the enhancement of the TB diagnosis strategies in late 2017, the bacteriological positivity rate of TB patients in Shenzhen increased significantly, but the problem of diagnosis delays was still serious. Active case-finding among risk populations could effectively reduce patient delay, and the application of molecular diagnostic technologies should be further promoted and optimized.

## Data availability statement

The original contributions presented in the study are included in the article/Supplementary material, further inquiries can be directed to the corresponding authors.

## Ethics statement

The studies involving human participants were reviewed and approved by the Ethics Committee of Shenzhen Chronic Disease Control and Prevention. Written informed consent from the patients/participants or patients/participants' legal guardian/next of kin was not required to participate in this study in accordance with the national legislation and the institutional requirements.

## Author contributions

Conceptualization, project administration, supervision, writing, review, and editing: QJ and W-GT. Data curation: C-YH, L-CJ, P-XL, and M-ZL. Data analysis: F-LW, Y-TZ, F-XT, and C-GY. Methodology: C-GY, W-GT, and QJ. Resources, software, visualization, and roles/writing original draft: C-YH and F-LW. All authors contributed to the article and approved the submitted version.
