# Supplementary material for: Time-trend analysis of tuberculosis diagnosis in Shenzhen, China between 2011 and 2020
Source: Front Public Health. 2023 Feb 20;11:1059433. doi: 10.3389/fpubh.2023.1059433 (PMC9986421; doi:10.3389/fpubh.2023.1059433)
Supplement: Supplementary file 1 [file Data_Sheet_1.PDF]

Supplementary tables for the manuscript “Time-trend Analysis of the Tuberculosis Diagnosis in Shenzhen, China During 2011-2020” by Chuang-Yue Hong, et al.

STable 1. Basic characteristics of the TB patients in Shenzhen during 2011-2020, stratified by the residence status

	Total (n=43,846)	Local resident (n=4,205)	Internal migrant (n=39,641)
Gender***			
Male	28730 (65.5)	2,465 (58.6)	13,376 (33.7)
Female	15116 (34.5)	1,740 (41.4)	26,265 (66.3)
Age***			
≤ 35 years	29,079 (66.3)	2,184 (51.9)	26,895 (67.8)
＞35 years	14,767 (33.7)	2,021 (48.1)	12,746 (32.2)
Nationality***			
Han	37,113 (84.6)	3,871 (92.2)	33,236 (83.8)
Minority	6,733 (15.4)	823 (7.8)	6,405 (16.2)
Occupation***			
Worker	14,897 (34.0)	692 (16.5)	14,205 (35.8)
Professional staff	5,897 (13.4)	1,692 (40.2)	4,187 (10.6)
Waiter	3,142 (7.2)	273 (6.5)	2,869 (7.2)
Unemployed	19,910 (45.4)	1,540 (36.6)	18,370 (46.3)
Patient delay**			
<30 days	30,541 (69.7)	2,844 (67.6)	27,697 (69.9)
≥30 days	13,305 (30.3)	1,361 (32.4)	11,944 (30.1)
Hospital delay***			
<14 days	30,218 (68.9)	2,648 (63.0)	27,570 (70.0)
≥14 days	13,628 (31.1)	1,557 (37.0)	12,071 (30.0)
Way of case-finding***			
Active	4,338 (9.9)	486 (11.6)	3,852 (9.7)
Passive	39,508 (90.1)	3,719 (88.4)	35,789 (90.3)
Bacteriological testing			
Negative	19,752 (45.0)	1,904 (45.3)	17,848 (45.0)
Positive	24,094 (55.0)	2,301 (54.7)	21,793 (55.0)
Severity			
No	39,405 (89.9)	3,783 (90.0)	35,622 (90.0)
Yes	4,441 (10.1)	422 (10.0)	4,019 (10.0)
Radiographic cavity***			
No	33,040 (75.4)	3,285 (78.1)	29,755 (75.1)
Yes	10,796 (24.6)	919 (21.9)	9,877 (24.9)
Drug resistance			
Sensitive	41923 (95.6)	4,003 (95.2)	37,920 (95.7)
Resistant	1923 (4.4)	202 (4.8)	1,721 (4.3)
TB history*			
New case	41,457 (94.6)	3,946 (93.8)	37,511 (94.6)
Retreated case	2,389 (5.4)	259 (6.2)	2,130 (5.4)
Treatment outcome			
Favorable	39,879 (91.0)	3,811 (90.6)	36,068 (91.0)
Unfavorable	3,693 (8.4)	351 (8.4)	3,342 (8.4)

Note: Statistical significance between local residents and internal migrants were tested by the chi-square test and marked with * <0.05; ** <0.01; ***<0.001.

STable 2. Between-group comparison of factors related to patient delay

	Patient delay (n=13,305)	Non-patient delay (n=30,541)	χ^2	*P*-value
Gender			107.58	< 0.0001
Female	5,062 (38.0)	10,054 (32.9)		
Male	8,243 (62.0)	20,487 (67.1)		
Age			305.69	< 0.0001
≤ 35	8,208 (60.9)	21,051 (68.9)		
> 35	5,277 (39.1)	9,490 (31.1)		
Ethnicity			18.83	< 0.0001
Han	11,413 (85.8)	25,700 (84.1)		
Minority	1,892 (14.2)	4,841 (15.9)		
Occupation			160.61	< 0.0001
Worker	4,132 (31.0)	10,765 (35.4)		
Unemployed	6,611 (49.6)	13,299 (43.7)		
Professional staff	1,581 (11.8)	4,298 (14.1)		
Waiter	978 (7.3)	2,164 (7.1)		
Census register			8.89	0.0029
Local resident	1,361 (10.2)	2,844 (9.3)		
Internal migrant	11,944 (89.8)	27,697 (90.7)		
Case finding			1,266.30	< 0.0001
Active case-finding	293 (2.2)	4,045 (13.2)		
Passive case-finding	13,032 (97.7)	26,496 (86.7)		
Bacteriological result			1,567.80	< 0.0001
Negative	4,113 (30.9)	15,639 (51.2)		
Positive	9,192 (69.1)	14,902 (48.8)		
Cavity			527.06	< 0.0001
No	9,074 (68.2)	23,966 (78.5)		
Yes	4,226 (31.7)	6,570 (21.5)		
Drug resistance			76.09	< 0.0001
Sensitive	12,549 (94.3)	29,374 (96.3)		
Resistant	756 (5.7)	1.167 (3.7)		
Treatment type			4.74	0.0295
New case	12,532 (94.2)	28,925 (94.7)		
Retreated case	773 (5.8)	1,616 (5.3)		
Treatment outcome			51.76	< 0.0001
Favorable	11,906 (89.5)	27,974 (91.6)		
Unfavorable	1,293 (9.7)	2,399 (7.9)		

STable 3. Between-group comparison of factors related to hospital delay

	Hospital delay (n=13,628)	Non-hospital delay (n=30,218)	χ^2	*P*-value
Gender			0.16	0.6925
Female	4,717 (34.6)	10,399 (34.4)		
Male	8,911 (65.3)	19,819 (65.5)		
Age			7.48	0.0062
≤ 35	9,164 (67.2)	19,915 (65.9)		
> 35	4,464 (32.7)	10,303 (34.0)		
Ethnicity			1.83	0.1766
Han	11,583 (84.9)	25,530 (84.4)		
Minority	2,045 (15.0)	4,688 (15.5)		
Occupation			309.65	<0.0001
Worker	4,553 (33.4)	10,344 (34.2)		
Unemployed	5,717 (41.9)	14,193 (46.9)		
Professional staff	2,394 (17.5)	3,485 (11.5)		
Waiter	956 (7.0)	2,186 (7.2)		
Census register			76.45	<0.0001
Local resident	1,557 (11.4)	2,648 (8.7)		
Internal migrant	12,071 (88.5)	27,570 (91.2)		
Case finding			2,439.50	<0.0001
Positive finding	2,778 (20.3)	1,560 (5.1)		
Passive finding	10,850 (79.5)	28,658 (94.8)		
Bacteriological result			2,892.50	<0.0001
Negative	8,733 (64.0)	11,019 (36.4)		
Positive	4,895 (35.9)	19,199 (63.5)		
Cavity			957.79	<0.0001
No	11,562 (84.8)	21,478 (71.0)		
Yes	2,063 (15.1)	8,733 (28.8)		
Drug resistance			117.58	<0.0001
Sensitive	13,246 (97.1)	28,677 (94.9)		
Resistant	382 (2.7)	1,541 (5.0)		
Treatment type			70.00	<0.0001
New case	13,070 (95.9)	28,387 (93.9)		
Retreated case	558 (4.0)	1,831 (6.0)		
Treatment outcome			25.44	<0.0001
Favorable	12,539 (92.0)	27,341 (90.4)		
Unfavorable	1,012 (7.4)	2,680 (8.8)		

STable 4. Risk factors for the patient delay and hospital delay by multivariable logistic regression, stratified by the intervention period

	Non-intervention period (before 2018)		Intervention period (2018-2020)	
	Patient delay aOR (95%CI)	Hospital delay aOR (95%CI)	Patient delay aOR (95%CI)	Hospital delay aOR (95%CI)
Female	1.35 (1.28-1.43)	-	1.23 (1.14-1.32)	-
Age > 35 years	1.30 (1.23-1.38)	-	1.47 (1.36-1.58)	-
Unemployed	1.27 (1.20-1.33)	0.86 (0.82-0.91)	-	0.94 (0.87-1.00)
Local resident	1.25 (1.14-1.38)	1.21 (1.09-1.34)	-	1.12 (1.01-1.24)
Retreatment	0.61 (0.54-0.69)	1.38 (1.19-1.61)	0.88 (0.76-1.02)	1.22 (1.05-1.41)
Passive case-finding	5.12 (4.47-5.89)	0.23 (0.22-0.25)	6.56 (5.08-8.64)	0.30 (0.26-0.35)
Bacteria positivity	2.34 (2.21-2.47)	0.20 (0.19-0.21)	1.49 (1.37-1.62)	0.65 (0.60-0.71)
Radiographic cavity	1.34 (1.26-1.42)	0.66 (0.61-0.71)	1.27 (1.16-1.38)	0.71 (0.65-0.78)
Drug resistance	1.25 (1.09-1.42)	-	-	0.83 (0.70-0.98)
Tested by molecular testing	-	-	1.21 (1.12-1.30)	0.54 (0.50-0.59)

Abbreviations: aOR, adjusted Odds Ratio; CI, Confidential Interval.

STable 5. Risk factors for the patient delay and hospital delay by multivariable logistic regression, stratified by the residence status

	Local resident		Internal migrant	
	Patient delay aOR (95%CI)	Hospital delay aOR (95%CI)	Patient delay aOR (95%CI)	Hospital delay aOR (95%CI)
Female	1.11 (0.96-1.27)	1.00 (0.87-1.14)	1.34 (1.28-1.40)	-
Age > 35 years	1.33 (1.16-1.53)	1.04 (0.91-1.19)	1.36 (1.29-1.42)	-
Unemployed	0.82 (0.67-1.00)	0.91 (0.75-1.11)	1.22 (1.17-1.28)	0.88 (0.84-0.92)
Retreatment	0.62 (0.47-0.82)	0.96 (0.72-1.27)	0.73 (0.66-0.80)	1.24 (1.11-1.38)
Passive case-finding	8.03 (5.53-12.14)	0.33 (0.27-0.41)	5.23 (4.61-5.96)	0.24 (0.22-0.26)
Bacteria positivity	1.58 (1.36-1.83)	0.65 (0.57-0.76)	2.01 (1.99-2.20)	0.30 (0.29-0.32)
Radiographic cavity	1.13 (0.92-1.38)	0.54 (0.43-0.68)	1.36 (1.29-1.43)	0.65 (0.61-0.69)
Drug resistance	0.99 (0.73-1.34)	0.96 (0.69-1.32)	1.13 (1.02-1.26)	0.87 (0.74-0.98)
Intervention period (2018-2020)	0.76 (0.66-0.88)	1.51 (1.31-1.74)	0.92 (0.87-0.97)	2.09 (1.99-2.21)

Abbreviations: aOR, adjusted Odds Ratio; CI, Confidential Interval.
